# The influence of socioeconomic factors on choice of infant male circumcision provider in rural Ghana; a community level population based study

**DOI:** 10.1186/s12887-017-0937-2

**Published:** 2017-08-29

**Authors:** Thomas Gyan, Kimberley McAuley, Natalie Strobel, Sam Newton, Seth Owusu-Agyei, Karen Edmond

**Affiliations:** 1 Division of Paediatrics, Faculty of Health and Medical Sciences, University of Western Australia, Level 4, Administration Building, Princess Margaret Hospital for Children, Perth, WA 6008 Australia; 2 Kintampo Health Research Centre, Ghana Health Service, Kintampo, Ghana; 3 School of Public Health, Kwame Nkrumah University of Science and Technology, Kumasi, Ghana; 4 United Nations Children’s Fund UNICEF, Kabul, Afghanistan

**Keywords:** Socio-economic, Infant, Male, Circumcision, Community, Population-based, Ghana

## Abstract

**Background:**

The influence of socio-economic determinants on choice of infant male circumcision provider is not known in areas with high population coverage such as rural Africa. The overall aim of this study was to determine the key socio-economic factors which influence the choice of infant male circumcision provider in rural Ghana.

**Methods:**

The study investigated the effect of family income, distance to health facility, and cost of the circumcision on choice of infant male circumcision provider in rural Ghana. Data from 2847 circumcised infant males aged under 12 weeks and their families were analysed in a population-based cross-sectional study conducted from May to December 2012 in rural Ghana. Multivariable logistic regression models were adjusted for income status, distance to health facility, cost of circumcision, religion, maternal education, and maternal age.

**Results:**

Infants from the lowest income households (325, 84.0%) were more likely to receive circumcision from an informal provider compared to infants from the highest income households (260, 42.4%) even after adjusting for religious affiliation (adjusted odds ratio [aOR] 4.42, 95% CI 3.12–6.27 p = <0.001). There appeared to be a dose response with increasing risk of receiving a circumcision from an informal provider as distance to a health facility increased (aOR 1.25, 95 CI 1.30–1.38 P = <0.001). Only 9.0% (34) of families in the lowest socio-economic quintile received free circumcision services compared to 27.9% (171) of the highest income families.

**Conclusions:**

The Government of Ghana and Non-Government Organisations should consider additional support to poor families so they can access high quality free infant male circumcision in rural Ghana.

## Background

Globally, male infants are circumcised mostly for medical and religious reasons [1, 2]. Male circumcision has been reported in a number of high quality trials to reduce human immunodeficiency virus (HIV) infection in adult males who live in communities with high HIV prevalence such as South and East Africa [3, 4]. Other health benefits are less clear though some families feel that it reduces risks of urinary tract infection and balanitis [1, 2]. Approximately 91% of infant (age 0–11 months) males are circumcised in Ghana [5] and other West African countries [2]. We reported high risks of concerning health care practices and morbidities following infant male circumcision in our community based study in rural Ghana [6]. Fifty eight percent of circumcisions were performed by informal providers; including Wanzams (village based traditional circumcision providers), family members, and drug sellers.

Initiatives to improve the health care practices of Wanzams and other circumcision providers are underway [7, 8]. These include training on infection control, instruments to perform circumcision and hygiene. However, other strategies to influence family’s care seeking patterns, improve use of health facilities, and improve use of trained circumcision providers are also needed. This requires an understanding of the key factors which influence a family’s choice of circumcision provider. A recent systematic review reported that socio-economic factors such as income, location (rural and urban), and cost of the circumcision were key determinants of choice of health service provider for infant male circumcision [2]. Socio-economic status, cost, and geographical access are also key determinants of care seeking for antenatal and birthing care in sub-Saharan African populations [9–14]. However, to our knowledge, there have been no studies from rural Africa that have investigated the effect of these factors on choice of infant male circumcision provider.

Thus, the overall aim of this study was to determine the key socio-economic factors which influence the choice of infant male circumcision provider in rural Ghana. The primary objective was to determine if socio-economic status was an important determinant of choice of circumcision provider. The secondary objectives were to assess the associations between distance to health facilities and cost of circumcision on choice of circumcision provider.

## Methods

### Study design and setting

This was a community level population-based cross-sectional study conducted in the Brong Ahafo Region of central Ghana from 21st May 2012 to 31st December 2012. Data were collected during a large neonatal vitamin A supplementation trial (Neovita) and full details are published elsewhere [15]. At the time of the circumcision study, 80% of the study population lived in rural settlements and almost 20% of mothers did not have primary school education. Four major district hospitals and 80 small health facilities provided health care services to the population. There were approximately 60 Wanzams and 100 formal circumcision providers (doctors, nurses, and medical assistants) at the time of the study.

### Data collection

All births in the Neovita study area were reported to the trial team via a network of fieldworkers and key informants. Fieldworkers visited all families at home between two hours and two days after birth and interviewed the mother of the infant, or the primary care giver. Fieldworkers weighed the baby and asked the mother or the primary care giver about: date of birth, site of birth, current address, distance to health facilities, socio-demographic characteristics, and socio-economic information (using an asset index). The fieldworkers also collected data on the vital status of the baby (including if the baby was alive, dead, or hospitalised).

Only male liveborn Neovita infants who were aged under 12 weeks were included to ensure the most accurate recall of circumcision related events. Infants were included in the Neovita trial if they were aged under three days, able to feed, were staying in the study area for at least six months after enrolment and their mother provided written informed consent. Follow-up visits were scheduled between eight to eleven weeks post birth and trained senior fieldworkers asked for consent to collect additional detailed data on: age at circumcision, site of circumcision, and type of circumcision provider. Infant male circumcision was supposed to be covered under the Ghana Health Insurance Scheme but it was well known that fees for circumcisions were charged by some formal and informal providers. So we also asked families if they had to pay any fees or “in-kind” contributions for the circumcision. Families were also asked if the study team could have access to the baby’s Neovita data including socio-economic, and socio-demographic data.

Fieldworkers were trained for two weeks in all study procedures prior to the commencement of the study. Interrater reliability was checked between all fieldworkers. During the study fieldworkers received scheduled and unscheduled supervisory visits from the study coordinator to assess data quality and consistency. The fieldworkers used standardised paper based data collection tools (including a standardised list of closed ended questions) for all interviews.

### Study definitions and categories

In our study a *‘formal circumcision provider’* was defined as a professionally trained, licensed, and regulated provider of circumcision services. This included: doctors, medical assistants, or nurses [2]. An ‘*informal circumcision provider’* was an untrained, unlicensed, unregulated private provider of circumcision services including: Wanzams (village based traditional circumcision providers), drug sellers, and family members [2, 8, 16]. To assess ‘*income status’* an asset index was constructed based on data collected on household assets (ownership of animals, television, motorcycle, etc) and housing material (walls, floor, windows, and roof). The index was analysed using principal component analysis (PCA) in Stata version 13 and categorised into five income quintiles [17]. ‘*Distance to a health facility’* was measured in kilometres using Geographic Information System (GIS) software and the most commonly used roads from each village to the nearest health facility. It was categorised into four levels (<1 km (kilometre), 1–4.9 km, 5–9.9 km, 10 km or more). Many of the families in our study had limited recall about the exact cash amounts they paid for their circumcision but could categorise their responses. Thus information on the exact cash amounts for ‘*cost of the circumcision’* was not collected and data were collected in the following categories: free, not free but less than 10 Ghana Cedis (Ghs), between 10 and 20 Ghs, 20 Ghs or more (at the time of conducting the study 1 Ghs = 0.6 United States dollars ($US)) [18]. *‘In kind contributions’* were defined as any non-cash payment to the formal or informal provider for the circumcision (e.g. bars of soap, chickens, kola nuts, and corn).

### Statistical analysis

Crude logistic regression models were used to examine the effect of income status on type of circumcision provider (informal vs formal). Odds ratios (ORs) and 95% confidence intervals (95% CI) were calculated. Multivariable logistic regression models were constructed apriori to adjust for the effect of important explanatory variables (income status, cost of circumcision, religion, maternal education, maternal age and distance to health facility). Model one assessed each of the infant and maternal characteristics as determinants of choice of informal provider, adjusting for income status, cost of circumcision, religion, maternal education and maternal age. Model two is the same as model one with an additional adjustment for distance to health facility. All analyses were conducted using STATA version 13.

We calculated that the 2800 infants included in this study would provide 80% power to detect at least a 20% effect due to income status on choice of circumcision provider. We assumed a 5% significance level and a baseline 60% risk of receiving circumcision from an informal circumcision provider [6].

### Ethical issues

Ethical approvals were obtained from Ghana Health Service Ethical Review Committee, the Institutional Ethics Committee of Kintampo Health Research Centre (KHRC), the Research Ethics Committee of London School of Hygiene and Tropical Medicine, and the Human Research Ethics Committee of the University of Western Australia. Written informed consent was obtained from all the families of the circumcised male infants.

### Role of funding source

The funders had no role in data gathering, data analysis, or writing of the report. The corresponding author had full access to all the data in the study, and for the decision to submit for publication.

## Results

**Fig. 1:**
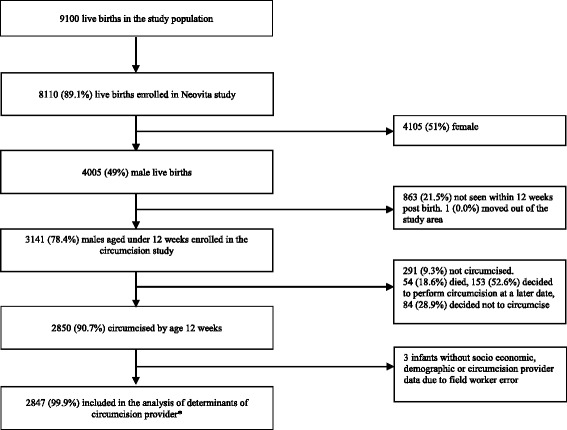
Flow diagram for the circumcision study. *Includes the 54 (18.6%) who died. These families were still interviewed and provided full information about circumcision thus their data were included

There were 9100 live births in the Neovita trial study area from 21st May to 31st December 2012 (Fig. 1). A total of 8110 (89%) liveborn infants were recruited into the Neovita study. Forty nine percent (4005) were male infants and 78% (3141) were aged under 12 weeks. Of the 3141 eligible male infants, 2850 (90.7%) were circumcised. Two hundred and ninety one (9.3%) infants were not circumcised within 12 weeks after birth. Of these, 153 (52.6%) were circumcised at a later date, 84 (28.9%) were never circumcised and 54 (18.6%) died. Three circumcised babies (0.1%) had no socio-economic or demographic data collected and were excluded in the statistical analysis of associations between socio-economic or demographic factors and choice of circumcision provider. Of the remaining 2847 circumcised infants, 1670 (59%) were circumcised by informal providers and 1177 (41%) by formal health service providers (Table 1). Three hundred and eighty seven (13.6%) were in the lowest socio-economic quintile, 186 (6.7%) lived 10 km or more from a health facility, and 512 (18.0%) mothers of circumcised infants had no primary school education (Table 1). A total of 666 (23.4%) mothers of circumcised infants were Muslim, and 549 (19.3%) delivered at home (Table 1). Five hundred and thirty nine (18.9%) infants received their circumcision free of charge (Table 2). A total of 2229 (78.3%) families paid some form of cash currency (between 1 and 100 Ghana Cedis (Ghs) [approximately 0.60 to 55 $US]) for their infant’s circumcision and 87 (3.1%) families paid in-kind contributions in the form of bars of soap, chickens, kola nuts, and corn (Table 3).

**Table 1:** Infant and maternal characteristics in the study population

Characteristics	Uncircumcised infants *n* = 291	Circumcised infants included in the analysis *n* = 2847 (99.9%)	Total circumcised infants *n* = 2850^a^
Income status of household (quintile)			
1 (Lowest)	115 (39.5%)	387 (13.6%)	387 (13.6%)
2	75 (25.8%)	532 (18.7%)	532 (18.7%)
3	47 (16.2%)	628 (22.1%)	628 (22.1%)
4	38 (13.0%)	687 (21.1%)	687 (24.1%)
5 (Highest)	16 (5.5%)	613 (21.5%)	613 (21.5%)
Missing data	–	–	3 (0.1%)
Distance to health facility			
< 1 km	101 (34.7%)	1444 (50.7%)	1444 (50.7%)
1–4.9 km	60 (20.6%)	741 (26.0%)	741 (26.0%)
5–10 km	84 (2.9%)	400 (14.0%)	400 (14.0%)
10 km or more	45 (15.5%)	186 (6.5%)	186 (6.5%)
Missing data	1 (0.3%)	76 (2.7%)	79 (2.8%)
Cost of circumcision^b^			
Free	–	539 (18.9%)	539 (18.9%)
Less than 10 Ghs	–	145 (5.1%)	145 (5.1%)
Between 10 and 20 Ghs	–	1530 (53.7%)	1530 (53.7%)
20 Ghs or more	–	554 (19.5)	554 (19.4)
Missing data	–	79 (2.8%)	82 (2.9%)
Maternal occupation			
Gov’t/private employed	4 (1.4%)	105 (3.7%)	105 (3.7%)
Self-employed	53 (18.2%)	927 (32.6%)	927 (32.5%)
Farming	101 (34.7%)	589 (20.7%)	589 (20.7%)
Not working	49 (16.8%)	484 (17.0%)	484 (17.0%)
Missing data	84 (28.9%)	742 (26.0%)	745 (26.4%)
Maternal highest educational level			
None	89 (30.6%)	512 (18.0%)	512 (18.0%)
Primary	138 (47.2%)	1481 (52.0%)	1482 (52.0%)
Secondary	63 (21.6%)	850 (29.9%)	852 (29.9%)
Missing data	1 (0.3%)	4 (0.1%)	4 (0.1%)
Religion			
Christian	186 (8.3%)	2048 (71.9%)	2048 (71.9%)
Muslim	61 (8.4%)	666 (23.4%)	666 (23.4%)
Traditional African/none	42 (24.6%)	129 (4.5%)	129 (4.5%)
Missing data	2 (0.7%)	4 (0.1%)	7 (0.2%)
Maternal age (years)			
Less than 20	41 (14.1%)	319 (11.2%)	319 (11.2%
20–29	145 (49.8%)	1458 (51.2%)	1458 (51.2%)
30 or more	104 (35.7%)	1066 (37.4%)	1066 (37.4%)
Missing data	1 (0.3%)	4 (0.1%)	7 (0.2%)
Site of delivery			
Health facility	187 (64.3%)	2292 (80.5%)	2292 (80.4%)
Home	101 (34.7%)	549 (19.3%)	549 (19.3%)
Missing data	3 (1.0%)	6 (0.2%)	9 (0.3%)
Birth weight			
Less than 2.5 kg	41 (14.1%)	214 (7.5%)	214 (7.5%)
2.5 kg or greater	250 (85.9%)	2633 (92.5%)	2636 (92.5%)
Missing data	–	–	–
Type of circumcision provider	–		
Formal provider	–	1177 (41.3%)	1177 (41.3%)
Informal provider	–	1670 (58.7%)	1670 (58.6%)
Missing data	–	0 (0.0%)	3 (0.1%)
Age at circumcision			
0–6 days	–	117 (4.1%)	117 (4.1%)
7–20 days	–	2556 (89.8%)	2556 (89.7%)
> 20 days	–	172 (6.0%)	172 (6.0%)
Missing data	–	2 (0.1%)	5 (0.2%)

^a^Three circumcised infants had no socioeconomic and demographic data due to field worker error

^b^1 Ghs = 0.6 $US (2012)

**Table 2:** Determinants of choice of informal provider for infant male circumcision

	Total number of infants	Number (%) of infants who received circumcision from an informal provider			
	*n* = 2847	*n* = 1670 (58.7%)	Unadjusted odds ratio (95% CI)	Adjusted odds ratio (95% CI) model 1^a^	Adjusted odds ratio (95% CI) model 2^b^
Income status of household (quintile)					
1 (Lowest)	387	325 (84.0%)	7.12 (5.19–9.76)	5.77 (4.15–8.02)	4.42 (3.12–6.27)
2	532	349 (65.6%)	2.59 (2.04–3.29)	2.26 (1.76–2.91)	1.89 (1.45–2.47)
3	628	355 (56.5%)	1.77 (1.41–2.21)	1.67 (1.32–2.11)	1.49 (1.17–1.90)
4	687	381 (55.5%)	1.69 (1.36–2.11)	1.58 (1.26–1.98)	1.48 (1.17–1.87)
5 (Highest)	613	260 (42.4%)	Ref	Ref	Ref
Missing data	0	–	–	-	-
Distance to health facility					
< 1 km	1444	776 (53.8%)	Ref	Ref	Ref
1–4.9 km	741	433 (58.4%)	1.21 (1.01–1.45)	1.20 (1.00–1.45)	1.20 (1.00–1.45)
5–9.9 km	400	286 (71.5%)	2.16 (1.70–2.74)	1.28 (1.00–1.67)	1.28 (1.00–1.67)
10 km or more	186	154 (83.4%)	4.41 (2.94–6.61)	2.70 (1.76–4.12)	2.70 (1.76–4.12)
Missing data	76	21 (26.6%)	–	-	-
		Mean 3.2 km sd 3.7	–	–	-
Cost of circumcision^c^					
Free	539	269 (49.9%)	Ref	Ref	Ref
Less than 10 Ghs	145	114 (78.6%)	3.69 (2.40–5.68)	2.43 (1.54–3.84)	2.41 (1.53–3.79)
Between 10 and 20 Ghs	1530	888 (58.0%)	1.39 (1.14–1.69)	1.22 (0.91–1.38)	1.14 (0.92–1.41)
20 Ghs or more	554	337 (60.8%)	1.56 (1.23–1.98)	1.32 (1.03–1.69)	1.26 (0.97–1.63)
Missing data	79	62 (78.5%)	–	–	–
Maternal occupation					
Gov’t/private employed	105	49 (46.7%)	0.63 (0.41–0.96)	1.19 (0.75–1.88)	1.41 (0.87–2.27)
Self-employed	927	514 (55.4%)	0.89 (0.71–1.11)	1.19 (0.92–1.54)	1.21 (0.93–1.57)
Farming	589	380 (64.6%)	1.31 (1.02–1.68)	0.91 (0.67–1.23)	0.87 (0.63–1.19)
Not working	484	282 (58.3%)	Ref	Ref	Ref
Missing data	742	445 (59.9%)	–	-	-
Maternal educational level					
None	512	359 (70.1%)	2.31 (1.83–2.92)	1.36 (1.05–1.77)	1.30 (1.00–1.70)
Primary	1481	882 (59.6%)	1.45 (1.23–1.72)	1.24 (1.04–1.48)	1.21 (1.01–1.45)
Secondary	850	427 (50.4%)	Ref	Ref	Ref
Missing data	4	2 (28.6%)	–	-	-
Maternal religion					
Christian	2048	1081 (52.9%)	Ref	Ref	Ref
Muslim	666	496 (74.5%)	2.60 (2.14–3.16)	2.26 (1.84–2.78)	2.40 (1.93–2.98)
Traditional African/None	129	91 (70.5%)	2.13 (1.45–3.15)	1.36 (0.90–2.04)	1.33 (0.88–1.99)
Missing data	4	2 (0.0%)	–	–	–
Maternal age (years)					
Less than 20	319	192 (60.4%)	Ref	Ref	Ref
20–29	1458	854 (58.6%)	0.93 (0.72–1.19)	1.10 (0.85–1.42)	1.05 (0.80–1.37)
30 or more	1066	622 (58.7%)	0.92 (0.72–1.19)	0.98 (0.74–1.28)	0.94 (0.71–1.23)
Missing data	4	2 (28.6%)	–	–	–
Site of delivery					
Health facility	2291	1241 (54.2%)	Ref	Ref	Ref
Home	549	425 (77.7%)	2.95 (2.37–3.66)	2.04 (1.62–2.59)	1.89 (1.49–2.41)
Missing data	6	4 (40.0%)	–	–	–
Birth weight					
Less than 2.5 kg	214	136 (63.6%)	1.25 (0.94–1.67)	1.24 (0.91–1.69)	1.24 (0.91–1.70)
2.5 kg or greater	2633	1534 (58.2%)	Ref	Ref	Ref
Missing data	0	0 (0.0%)	–	–	–
Age at circumcision					
0–6 days	117	68 (58.1%)	Ref	Ref	Ref
7–20 days	2556	1483 (58.3%)	0.99 (0.68–1.45)	1.09 (0.74–1.62)	1.08 (0.71–1.64)
> 20 days	172	119 (69.2%)	1.62 (0.99–2.64)	1.31 (0.78–2.21)	1.29 (0.75–2.22)
Missing data	2	0 (0.0%)	–	–	–

*Ref* Reference group, *CI* Confidence interval, *sd* Standard deviation

^a^ Model 1. Adjusted for income status, cost of circumcision, religion, maternal education and maternal age

^b^Model 2. Further adjusted for distance to health facility

^c^1 Ghs = 0.6 $US (2012)

**Table 3:** Details of cash payments and in-kind payments by provider type

Cost of circumcision^a^	Provider Type															
	All providers		Doctor		Medical assistant		Nurse		Drug seller		Domestic helper		Wanzam		Other^b^	
	Total	Included in-kind payment	Total	Included in-kind payment	Total	Included in-kind payment	Total	Included in-kind payment	Total	Included in-kind payment	Total	Included in-kind payment	Total	Included in-kind payment	Total	Included in-kind payment
	n(%)	n(%)	n(%)	n(%)	n(%)	n(%)	n(%)	n(%)	n(%)	n(%)	n(%)	n(%)	n(%)	n(%)	n(%)	n(%)
Total	2847	87 (3.1%)	136 (4.8%)	1 (0.7%)	83 (2.9%)	0 (0.0%)	958 (33.6%)	2 (0.2%)	175 (6.1%)	6 (3.4%)	454 (15.9%)	7 (1.5%)	979 (34.4%)	72 (7.4%)	62 (2.2%)	0 (0.0%)
Free	539 (18.9%)	16 (3.0%)	81 (59.5%)	1 (1.2%)	34 (41.1%)	0 (0.0%)	155 (16.2%)	1 (0.6%)	16 (9.1%)	2 (12.5)	183 (40.3%)	3 (1.6%)	68 (6.9%)	10 (14.0%)	2 (3.2%)	0 (0.0%)
1.00–9.9 Ghs	145 (5.1%)	4 (2.9%)	4 (2.9%)	0 (0.0%)	3 (3.6%)	0 (0.0%)	24 (2.5%)	0 (0.0%)	11 (6.2%)	0 (0.0%)	13 (2.9%)	0 (0.0%)	84 (8.6%)	4 (4.6%)	1 (1.6%)	0 (0.0%)
Between 10 and 20 Ghs	1530 (53.7%)	42 (2.8%)	17 (12.5%)	0 (0.0%)	29 (34.9%)	0 (0.0%)	596 (62.2%)	0 (0.0%)	122 (69.0%)	2 (1.6%)	154 (33.9%)	2 (1.3%)	579 (59.0%)	38 (6.6%)	33 (53.2%)	0 (0.0%)
20.00–100.00 Ghs	554 (19.5%)	23 (4.2%)	31 (22.8%)	0 (0.0%)	16 (19.3%)	0 (0.0%)	170 (17.7%)	1 (0.6%)	23 (13.1%)	2 (8.7%)	100 (22.0%)	2 (2.0%)	188 (19.2%)	18 (9.6%)	26 (41.9%)	0 (0.0%)
Missing data	79 (2.8%)	2 (2.5%)	3 (2.2%)	0 (0.0%)	1 (1.2%)	0 (0.0%)	13 (1.4%)	0 (0.0%)	3 (1.7%)	0 (0.0%)	4 (0.9%)	0 (0.0%)	55 (5.6%)	2 (3.6%)	0 (0.0%)	0 (0.0%)

^a^1 Ghs = 0.6 $US (2012)

^b^Other: These included family members and friends

Infants from the lowest income households (quintile 1) (325, 84.0%) were four times more likely to receive a circumcision from an informal provider compared to infants from the highest income households (260, 42.4%) (adjusted odds ratio [aOR] 4.42, 95% CI 3.12–6.27 p = <0.001) (Table 2). There also appeared to be a ‘dose response’ with increasing risk of receiving a circumcision from an informal provider as income status decreased (Table 2) (aOR 1.34, 95% CI 1.25–1.43 *p* = <0.001).

A total of 2229 (78.3%) families paid to receive circumcision services from both formal and informal circumcision providers (Tables 2 and 3). Five hundred and thirty nine (18.9%) infants received their circumcision free of charge (50.1% formal and 49.9% informal) (Tables 2 and 3). Only 6.9% (68) of Wanzams provided free circumcisions. In contrast, 59.5% of circumcisions were provided free by doctors, 16.2% by nurses, 41.1% by medical assistants, 9.1% by drug sellers, and 40.3% by domestic helpers (Table 3).

Families in the lowest income quintile also appeared to be the least likely to receive free circumcision services (Table 4). Only 9.0% of families in the lowest quintile received free circumcision services compared to 27.9% in the highest quintile (aOR 0.40, 95% CI 0.28–0.58 *p* = <0.001). There also appeared to be a ‘dose response’ where the likelihood of receiving a free circumcision decreased as income status decreased (aOR 0.35, 95% CI 0.23–0.53 *p* = <0.001). 58.7% of families in the lowest quintile paid between 10 and 20 Ghana Cedis for their circumcision and 20.2% paid 20–100 Ghana Cedis.

**Table 4:** Cost of circumcision by household income status

		Cost of circumcision^b^				
Household income status^a^	Total	Free	Less than 10 Ghs	Between 10 and 20 Ghs	20.00–100 Ghs	Missing data
	*n* = 2847	*n* = 539	*n* = 145	*n* = 1530	*n* = 554	*n* = 79
		n (%)	n (%)	n (%)	n (%)	n (%)
1 (Lowest)	387	34 (9.0%)	41 (10.6%)	227 (58.7%)	78 (20.2%)	7 (1.8%)
2	532	55 (10.3%)	27 (5.0%)	319 (60.0%)	118 (22.2%)	13 (2.4%)
3	628	114 (18.2%)	25 (4.0%)	367 (58.4%)	104 (16.6%)	18 (2.9%)
4	687	165 (24.0%)	34 (4.9%)	336 (48.9%)	132 (19.2%)	20 (2.9%)
5 (Highest)	613	171 (27.9%)	18 (2.9%)	281 (45.8%)	122 (19.9)	21 (3.4%)

^a^Weath quintile calculated using principal components analysis

^b^1 Ghs = 0.6 $US (2012)

Eighty seven (3.1%) families paid in-kind contributions in the form of bars of soap, chickens, kola nuts, and corn (Table 3). The payment of in-kind contributions was more common with Wanzams (7.4%) than doctors (0.7%), nurses (0.2%), medical assistants (0.0%), drug sellers (3.4%), and domestic helpers (1.5%) (Table 3). Families of low socio-economic status appeared to be more likely to pay additional in-kind contributions (31.0%) compared to highest income families (11.5%) (aOR 0.41, 95% CI 0.25–0.67 *p* = <0.001).

Infants who lived 10 km or more from a health facility (154, 83.4%) were two times more likely to receive their circumcision from an informal provider compared to infants who lived less than 1 km from a health facility (776, 53.8%) (aOR 2.70, 95% CI 1.76–4.12 *p* = <0.001) (Table 2). There also appeared to be a dose response with increasing risk of receiving a circumcision from an informal provider as distance to a health facility increased (Table 2) (aOR 1.25, 95 CI 1.30–1.38 *P* = <0.001).

Household income status was closely associated with distance to a health facility (Table 5). Families in the lowest socio-economic quintile lived an average of 6.1 km (standard deviation [sd] 4.4 km) from a health facility (median 6.9 km, interquartile range [IQR] 1–15.9 km) compared to an average of 1.1 km in families in the highest socio-economic quintile (sd 1.6 km, median 0.6 km, IQR 0–10.4 km) (Table 5). Families in the lowest socio-economic quintile (79, 42.5%) were 22 times more likely to live more than 10 km from a health facility compared to families in the highest socio-economic quintile (5, 2.7%) (aOR 22.35 95% CI 8.84–56.54 *p* = <0.001) (Table 5). However, both socio-economic status (aOR 1.32, 95 CI 1.23–1.41 *P* = <0.001) and distance to health facilities (aOR 1.28, 95 CI 1.14–1.43 *P* = <0.001) had independent effects on the choice of circumcision provider.

**Table 5:** Distance to health facility by household income status

Household income status	Total	Mean distance (sd)	Median distance (interquartile range)	Min. & max. Values	< I Km	1–4.9 Km	5–9.9 Km	10 Km or more	Not known/missing
	n (%)	km	Km	km	n (%)	n (%)	n (%)	n (%)	n (%)
Total	2847	3.1 (3.6)	1.1 (0–12.8)	0 & 18.9	1444	741	400	186	76
1 (Lowest)	387 (13.6%)	6.1 (4.4)	6.9 (1–15.9)	0 & 18.5	106 (7.3%)	49 (6.1%)	153 (38.3%)	79 (42.5%)	0 (0.0%)
2	532 (18.7%)	3.9 (3.8)	2.1 (0–12.3)	0 & 13.1	218 (15.1%)	117 (15.8%)	132 (33.0%)	55 (29.6%)	10 (13.2%)
3	628 (22.1%)	2.4 (3.0)	0.9 (0–11.3)	0 & 12.3	321 (22.2%)	203 (27.4%)	62 (15.5%)	35 (18.8%)	7 (9.2%)
4	687 (24.1%)	1.7 (2.2)	0.8 (0–10.9)	0 & 12.7	387 (26.8%)	228 (30.8%)	42 (10.5%)	12 (6.5%)	18 (23.7%)
5 (Highest)	613 (21.5%)	1.1 (1.6)	0.6 (0–10.4)	0 & 11.4	412 (28.5%)	144 (19.4%)	11 (2.8%)	5 (2.7%)	41 (53.9%)

There was no statistical evidence of modification of the effect of distance from health facility on the choice of provider for circumcision by income status of the household (*p*-value for the interaction, 0.188).

Infants were two times more likely to receive circumcision from an informal provider if the families were Muslim (496, 74.5%) compared to Christian (1081, 52.9%) (aOR 2.40, 95% CI 1.93–2.98 *p* = <0.001) (Table 2). Mothers with no formal education (359, 70.1%) were 30% more likely to receive an informal circumcision provider compared to mothers with secondary level education (427, 50.4%) (aOR 1.30, 95% CI 1.00–1.70 *p* = <0.049) even after adjusting for other variables. There were no obvious differences associated with other socio demographic characteristics (Table 2).

## Discussion

In our population-based study in rural Ghana, infant male circumcision was almost universal (91%) and was performed by both formal (41%) and informal (59%) circumcision providers. Both socio-economic status and geographic access to health facilities had important and independent effects on the choice of circumcision provider. The risk of receiving a circumcision from an informal provider increased with each level of deprivation and with the distance that families lived from health facilities. We also found that families with the lowest household income were the most likely to pay for their circumcision. Poor families were also most likely to pay additional in-kind contributions.

The relationship between socio-economic status [2, 19–21], geographic access [2, 22, 23], and choice of informal provider for infant male circumcision has been reported in many studies in low and middle income countries. However, our study is the first to report data from a rural area in Africa with high population level coverage of infant male circumcision. This is also the first study to report the double burden that circumcision places on families of low socio-economic status. In our study poor families were more likely to receive a circumcision from an untrained informal provider and also more likely to incur a significant economic cost.

In 2008, the “Free Maternal Care Policy” [24] was introduced into the Ghana Health Insurance Scheme [25]. Under the policy, all pregnant women and their infants up to 90 days postpartum and all children aged 90 days to 18 years are meant to receive free care in accredited public and private healthcare facilities. The services that are covered include antenatal care, delivery care, postnatal care, and infant male circumcision. Mothers and children just have to be registered and receive a registration card. The registration process is free and there are meant to be no out of pocket expenses. However, there have been difficulties in enrolling many families into the scheme. This has been attributed to difficulties in accessing many areas of Ghana, especially the poorest and most disadvantaged areas [26, 27]. In 2011, close to the time of conducting this study, only 33% of Ghana’s population were registered with 4.2% coverage for the poorest [27]. The most recent data from 2013 indicate that the national coverage still remains limited with only 38% registered [28]. Inequity in health insurance coverage is likely to be an important driver of the costs of circumcision incurred by poor families that we reported. Our study area is located in central rural Ghana in the Brong Ahafo region and health insurance coverage in the Brong Ahafo region was 45.9% in 2011 [27]. However, there are no data on coverage of health insurance in the poorest families in our study area.

Antenatal care and delivery services are also meant to be free under the Ghana health insurance scheme [24, 25] and similar inequities are also reported for these services. There are reports of poor women being charged unofficial and non-legitimate fees for delivery and postnatal care services [29] (https://www.ghanabusinessnews.com/2016/04/23/ghanas-free-maternal-healthcare-policy-not-workingresearch/). Reports of poor women and their babies being forcibly kept in birthing hospitals until their bills are settled have also been published [29]. Poor women have also been charged unofficial fees for antenatal (http://vibeghana.com/2012/01/18/free-maternal-health-policy-is-it-really-working/), delivery, and postnatal care services (http://www.ghanavoice.com/2016/04/23/ghanas-free-maternal-healthcare-policy-not-working-research/) in accredited facilities because they were unable to confront authority figures [30, 31]. Poor women are also less likely to be insured for delivery care compared to richer women in Ghana [32, 33].

Additional economic costs of circumcision include the payment of ‘in-kind’ contributions. The payment of in-kind contributions was more common with Wanzams (7%) than formal providers (3%) (doctors, nurses, and medical assistants) in our study. The poorest families also paid more in-kind contributions (31%) than the highest income families (12%). Two rural Kenyan studies have reported the payment of in-kind contributions (chickens, sheep, food and medical supplies) by families for circumcision [21, 22]. In these studies medical practitioners (49%) and informal traditional providers (51%) received similar in-kind contributions. However, these studies did not provide any information on the in-kind contributions paid by poor and richer families within the same study area.

We also reported that families of the Muslim religion were two-fold more likely to choose an informal provider than families with other religious affiliations. The Muslim religion is a well-known determinant of use of informal providers for circumcision in urban and rural Africa [2, 19, 34] and many Wanzams are Muslim themselves [8]. Approximately, 70% of Wanzams who performed circumcisions in our rural study area were Muslims. We also reported that mothers with no formal education were more likely to choose an informal circumcision provider compared to mothers with secondary level education. These data are also consistent with other African studies [35]. There were no obvious differences in choice of circumcision provider associated with other socio-demographic characteristics in our study.

Our study had some limitations. Investigators from Egypt have reported a lack of confidence in the formal health care system as a reason for the use of informal circumcision providers who charge fees [2, 36]. These studies also reported that traditional providers were perceived as more experienced and better in providing healthcare than formal health service providers [36]. However, we were not able to conduct indepth qualitative interviews to explore perspectives and experiences of families and health service providers in our study. We were also unable to assess family’s perceptions of quality of care. We were also unable to collect data on transport costs and other opportunity costs incurred by the families. Our study was observational and cross-sectional and does not provide proof of causation. However, we controlled for a wide range of individual, household and community level confounders and strengths of our study included its large community and population-based data collection system. In addition 22% of babies were not able to be visited within a 12 week period after birth. Anecdotal information from the study area indicated that these families needed to travel more for employment and they were of lower socio economic status and educational levels. The omission of these infants reduces the generalisability of our study a little but is unlikely to have introduced any systematic bias.

## Conclusions

Our study appears to be the first to analyse the “on the ground” “community level” influence of socioeconomic factors on choice of infant male circumcision provider in an area with almost total population coverage. It also appears to be the first study that has described the high and inequitable costs paid by the poorest families in rural Africa for infant male circumcision. The Government of Ghana and other Non-Government Organisations should provide additional support to poor families so they can access high quality free infant male circumcision in rural Ghana. This includes improved coverage of Ghana’s free maternal care policy and health insurance scheme for the poorest families.

## Abbreviations

$US: United States dollars
AOR: Adjusted odds ratio
CI: Confidence interval
Ghs: Ghana Cedis
GIS: Geographic information system
HIV: Human immunodeficiency virus
IQR: Interquartile range
KHRC: Kintampo Health Research Centre
KM: Kilometre
ORs: Odds ratios
PCA: Principal component analysis
Ref: Reference group
SD: Standard deviation
